# Development of Lipid–Polymer Hybrid Nanoparticles for Improving Oral Absorption of Enoxaparin

**DOI:** 10.3390/pharmaceutics12070607

**Published:** 2020-06-30

**Authors:** Bo Tang, Yu Qian, Guihua Fang

**Affiliations:** 1 School of Pharmacy, Nantong University, 19 Qixiu Road, Nantong 226001, China; tangbo@ntu.edu.cn (B.T.); syfsxyhh18@163.com (Y.Q.); 2 School of Pharmacy, Shenyang Pharmaceutical University, 103 Wenhua Road, Shenyang 110016, China; 3 Yabang Medical Research Institute, 66 Changhong Road, Changzhou 213145, China

**Keywords:** enoxaparin, lipid–polymer hybrid nanoparticles, oral, intestinal absorption

## Abstract

Enoxaparin, an anticoagulant that helps prevent the formation of blood clots, is administered parenterally. Here, we report the development and evaluation of lipid–polymer hybrid nanoparticles (LPHNs) for the oral delivery of enoxaparin. The polymer poloxamer 407 (P407) was incorporated into lipid nanoparticles to form gel cores and ensure high encapsulation efficiency and the controlled release of enoxaparin. In vitro results indicated that 30% of P407 incorporation offered higher encapsulation efficiency and sustained the release of enoxaparin. Laser confocal scanning microscopy (LCSM) images showed that LPHNs could not only significantly improve the accumulation of enoxaparin in intestinal villi but also facilitate enoxaparin transport into the underlayer of intestinal epithelial cells. In vivo pharmacokinetic study results indicated that the oral bioavailability of enoxaparin was markedly increased about 6.8-fold by LPHNs. In addition, its therapeutic efficacy against pulmonary thromboembolism was improved 2.99-fold by LPHNs. Moreover, LPHNs exhibited excellent biocompatibility in the intestine. Overall, the LPHN is a promising delivery carrier to boost the oral absorption of enoxaparin.

## 1. Introduction

Heparin is an anticoagulant that prevents the formation of blood blots, and it has shown great prevention and therapeutic efficacy in terms of deep-vein thrombosis (DVT), pulmonary embolism (PE), and venous thrombosis [1] clinically. Low molecular weight heparin (LMWH) is obtained from unfractionated heparin (UFH) by chemical and enzymatic depolymerization [2]. Enoxaparin, one of the most commonly used LMWH, holds a longer half-life in vivo than UFH, which reduces the administration frequency [3]. However, its oral absorption is still low due to its large molecular weight, high anionic charges, and first-pass effect in the liver [4,5,6]. Therefore, it is administered via the parenteral route, which is less convenient and has lower compliance for patients. To translate the administration route from injection to oral delivery, it is quite crucial to increase the oral absorption of LMWH [7,8]. With the advances in nanotechnology, polymer- and lipid-based nanocarriers such as polymeric micelles [9], polymeric nanoparticles [10], lipid nanocapsules [11], microemulsions [12], and solid lipid nanoparticles [13] have been intensively used to facilitate the oral absorption of LMWH. Rationally designed nanocarriers are able to overcome the hurdles encountered during the absorption process through following ways, including (1) protecting drugs from acidic degradation in the stomach; (2) increasing the intestinal epithelial permeability; (3) facilitating intestinal lymphatic transport [13,14,15,16,17,18].

Solid lipid nanoparticles (SLNs) are colloidal drug delivery systems consisting of surfactant-stabilized lipids that are solid both at room and body temperature [13]. They integrate the advantages of liposomes, polymeric nanoparticles, and emulsions. In addition, SLNs possess a solid lipid core matrix, so they are used to encapsulate lipophilic drugs in most cases [19,20]. Because LMWH is hydrophilic, the encapsulation efficiency of LMWH in the SLNs is low, which leads to insufficient therapeutic concentration in vivo. This is due to hydrophilic drugs having limited loading quantity and homogeneity in the lipid cores. To improve the encapsulation efficiency of LMWH in the SLNs, conjugating lipidic molecules with LMWH via chemical synthesis was reported in a previous study [13]. Although the oral absorption of LMWH is significantly improved in this way, there may be some problems associated with the chemical modification of LMWH. Since LMWH exerts its therapeutic effects by binding to antithrombin III (AT III) via a unique pentasaccharide motif [21], chemical synthesis may increase the risk of reducing or losing the activity of LMWH. To avoid the possibility of reducing or losing drug activity, a common alternative strategy is to prepare LMWH-loaded SLNs by a double emulsion (water-in-oil-in-water, W/O/W) method. However, the encapsulation efficiency of LMWH in the SLNs is still unsatisfactory. Therefore, a new encapsulation strategy is needed to further improve the encapsulation efficiency of LMWH.

It has been reported that hydrophilic viscosity-enhancing agents such as propylene glycol (PG) and polyethylene glycol (PEG) 400 and PEG 600 are able to increase the encapsulation efficiency of insulin in the SLNs [22]. Inspired by this, hydrogels may be an alternative. Hydrogels, a network of polymer chains, are often used for the delivery of hydrophilic drugs with higher drug loading [23]. Poloxamer 407 (P407) is a triblock copolymer consisting of a central hydrophobic block and two hydrophilic blocks of polyethylene glycol at both ends. P407-based hydrogels exhibit interesting nature at certain concentration levels. That is, they are in a liquid state below gelation temperature and turn into a viscosity-enhancing gel above gelation temperature [24,25].

Encouraged by the advantage of SLNs and hydrogels, we attempted to fabricate lipid–polymer hybrid nanoparticles (LPHNs) for the oral delivery of enoxaparin. In this study, poloxamer 407 is used to improve the encapsulation efficiency and control the release of enoxaparin. The lipid–polymer hybrid nanoparticles were characterized in terms of size and zeta potential, encapsulation efficiency, and particle morphology. In vitro release behavior was also investigated. The intestinal absorption was evaluated by laser confocal scanning microscopy. In addition, in vivo absorption, in vivo efficacy, and safety tests were performed by rat experiments. In all, we attempt to investigate whether lipid–polymer hybrid nanoparticles can increase encapsulation efficiency and boost the oral absorption of enoxaparin.

## 2. Materials and Methods

### 2.1. Materials

Enoxaparin (mean MW 4251 Da, 101 IU/mg) was purchased from Hangzhou Jiuyuan Gene Engineering Co., Ltd. (Hangzhou, China). Precirol ATO 5 (glyceryl palmitostearate) was kindly donated by Gattefosse (Lyon, France). Egg yolk lecithin (E80) was obtained from Lipoid KG (Ludwigshafen, Germany), and Tween 80 was purchased from BASF (Ludwigshafen, Germany). Poloxamer 407 (BASF, Ludiwigshafen, Germany) was purchased from Xi’an Yuelai Medical Technology Co., Ltd. (Xi’an, China). Fluorescein isothiocyanate (FITC) was obtained from Shanghai Golden Wheat Biotechnology Co., Ltd. (Shanghai, China). Tissue-Tek O.C.T. compound (SAKURA, Torrance, CA, USA) was purchased from Nantong Qixiang Biotechnology Co., Ltd. (Nantong, China). Activated partial thromboplastin time (APTT) assay kits were obtained from Nanjing Caobenyuan Biotechnology Co., Ltd. (Nanjing, China). All other chemicals were of analytical grade.

### 2.2. Preparation of Lipid–Polymer Hybrid Nanoparticles (LPHNs)

Enoxaparin-loaded LPHNs were prepared as follows. In brief, 12.5 mg of enoxaparin was dissolved in 0.5 mL-differentiated ratios of poloxamer 407 (P407) aqueous solution at 4 °C. Then, 10 mg E 80 and 40 mg Precirol ATO 5 were dissolved in 2 mL dichloromethane (DCM). DCM was dropped into 0.2 mL of P407 aqueous solution containing enoxaparin. Then, this mixed solution was ultrasonicated using a probe sonicator (Ningbo Xinzhi Biological Technology Co. Ltd., Ningbo, China) for 2 min at 500 W to obtain primary W/O emulsion. Subsequently, 2% Tween 80 aqueous solution added to the obtained primary emulsion followed by ultrasonication for 1 min at 380 W. Finally, the obtained formulation was transferred into a flask to remove the DCM at 34 °C, using a rotary evaporator (Eyela, Tokyo, Japan). The preparation method for enoxaparin-loaded SLNs was the same as that for enoxaparin-loaded LPHNs, except that no P407 was in the aqueous solution.

### 2.3. In Vitro Characteristics

#### 2.3.1. Size, Zeta Potential, and Encapsulation Efficiency

The particle size and size distribution of prepared nanoparticles were measured by 90 plus zeta (Brookhaven, MS, USA) at room temperature. The zeta potential of nanoparticles was tested using the 90 plus zeta by electrophoretic laser doppler anemometry at room temperature. All the samples were diluted with deionized water, and measurements were taken in triplicate.

The encapsulation efficiency of enoxaparin in nanoparticles was determined by an ultra-filtration method [26]. An appropriate amount of nanoparticle dispersion was added in a Millipore Amicon® Ultra filteration tube (MWCO: 100 kDa). Free enoxaparin was separated from the nanoparticle dispersion by centrifugation at 2000 rpm for 15 min. To determine the total amount of the drug, including the free drug in the dispersion and encapsulated drug in the nanoparticles, an appropriate amount of nanoparticle dispersion was destroyed by DCM, and the released enoxaparin was extracted by deionized water. The enoxaparin in the ultrafiltrate and nanoparticle dispersion was determined by the Azure II colorimetric method using a multimode microplate reader (Bio Tek, Winooski, VT, USA) at 606 nm [27]. The linearity range of this method was determined between 0 and 6 μg/mL, with a linear correlation coefficient of 0.9973. All samples were measured in triplicate. The encapsulation efficiency (EE) of enoxaparin was calculated using the following equation:

(1) $$EE\%=\frac{W_{totaldrug}-W_{freedrug}}{W_{totaldrug}}$$

where *W*_total drug_ is the total amount of drug in the nanoparticle dispersion, and *W*_free drug_ is the total amount of drug in the ultrafiltrate.

In addition, the prepared nanoparticle suspension was placed at 4 °C in a refrigerator for 1 week to determine whether the encapsulation efficiency changes with time.

#### 2.3.2. Particle Morphology

The morphology of nanoparticles was examined by transmission electron microscopy (TEM). Samples of nanoparticles were diluted with deionized water, dropped onto a copper grid, and then stained with phosphotungstic acid. The samples were subjected to TEM (JEOL, Tokyo, Japan) after drying.

#### 2.3.3. In Vitro Drug Release

In vitro release of enoxaparin from the nanoparticles was studied using the dialysis method, and an enoxaparin solution was used as control. Briefly, 2 mL of nanoparticle suspension was transferred into dialysis bags (Biosharp Biotechnology Co. Ltd., Hefei, China, MWCO: 14 kDa) and dialysis bags were immersed into a beaker containing 25 mL pH 6.8 phosphate buffer. Then, the beaker was placed in a 37 °C water bath with a magnetic stirring speed of 150 rpm. At a predetermined time point, the medium in the beaker was withdrawn, followed by replacement with the same volume of fresh release medium. The released enoxaparin content was determined by the Azure II colorimetric method, as mentioned in the previous part.

### 2.4. Intestinal Absorption

To investigate the intestinal absorption of nanoparticles, in vivo experiments were conducted in rats. All animal studies were conducted according to the guidelines of the local Institute Animal Ethical Care Committee (IAEC, 20180512-003). To visualize the intestinal absorption, fluorescein isothiocyanate (FITC) was used to label enoxaparin. FITC was conjugated with enoxaparin according to the method previously described [28]. Briefly, 2 mg of FITC dissolved in dimethylsulfoxide was slowly added to 0.1 M sodium carbonate, and then added in 50 mg of enoxaparin. The reaction was performed in a ice-water bath, with a stirring speed of 150 rpm in the dark. After 8 h, the reaction was stopped by adding an ammonium chloride solution. Then, the resulting FITC-enoxaparin conjugate was introduced into a dialysis bag (MWCO: 1000 Da) to remove the byproduct. The dialyzed product was lyophilized at −50 °C to obtain FITC-labeled enoxaparin. Male SD rats (200 ± 20 g) were given FITC-enoxaparin solution and FITC-enoxaparin LPHN2 by gavage at a dosage of 505 IU/kg. After 30 min, the rats were sacrificed, and then the jejunum was removed, washed, and fixed with 4% paraformaldehyde for 4 h at room temperature, and dehydrated with 20% sucrose solution. The segments were frozen in cryo-embedding media, sectioned at 20 μm, and placed on polysine-coated slides. The sections were fixed with 4% paraformaldehyde for 30 min and rinsed three times with pH 7.4 phosphate buffer, and the intestine sections were then incubated with 20 μL of 4′,6-diamidino-2-phenylindole (DAPI) for 10 min in the dark to stain the nucleus, followed by mounting with antifluorescence quenching reagent. Finally, the sections were observed under a Leica SP8 laser confocal scanning microscope.

### 2.5. In Vivo Pharmacokinetic Study in Rats

In vivo absorption of nanoparticles was studied in rats. Male SD rats (200 ± 20 g) were divided randomly into three groups, with four rats per group. After fasting for 24 h, the rats were given enoxaparin solution and enoxaparin-loaded LPHN2 by gavage at a dose of 1010 IU/kg. At predetermined time intervals (0, 1, 3, 5, 8, 12 h), blood samples (about 0.5 mL) were drawn from the rats. Plasma was obtained by centrifugation (6000 rpm, 10 min) and analyzed by measuring the activated partial thromboplastin time (APTT) value according to a standard commercial kit. The absolute bioavailability (F) of orally administered formulations was calculated by comparing their AUC with that intravenous injection of enoxaparin solution (101 IU/kg).

### 2.6. In Vivo Efficacy in Mice

The in vivo prevention of pulmonary thromboembolism of nanoparticles was studied in mice. Male Kunming mice (18~22 g) were divided randomly into four groups, with 12 mice per group. Two groups were treated with enoxaparin solution and enoxaparin-loaded LPHN3 via intragastric administration at a single dosage of 1010 IU/kg, respectively. Two groups were given 100 μL of enoxaparin solution (101 IU/kg) and saline as control. Two hours after administration, all groups were intravenously injected with 100 μL of 1250 IU/kg thrombin to induce hind limb paralysis or death. The number of dead or paralyzed mice was recorded within 20 min; the results are shown as a percentage of protection.

### 2.7. Safety Evaluation

To investigate whether nanoparticles cause intestinal membrane damage or not, a histopathological examination was conducted. In this experiment, enoxaparin-loaded nanoparticles were given orally to rats at 1010 IU/kg, and physiological saline was given orally as a control. Then, the rats were sacrificed after 2 and 8 h. The jejunum was removed from rats and placed in 5% formaldehyde solution and stained with hematoxylin-erosin for histological studies.

### 2.8. Statistical Analysis

Statistical analysis was performed using a Student’s *t*-test. Data were expressed as mean ± SD. Statistical significance was represented by * *p* < 0.05 and ** *p* < 0.01.

## 3. Results and Discussion

### 3.1. Preparation and Characterization of Lipid–Polymer Hybrid Nanoparticles

**Figure 1:**
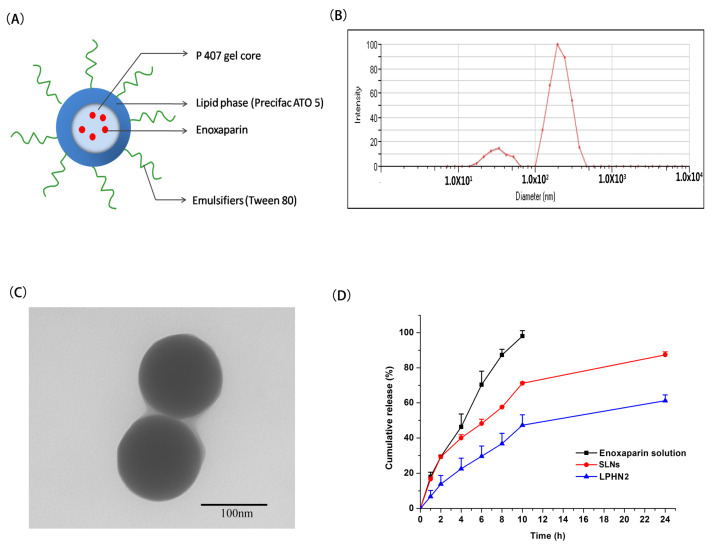
(**A**) Schematic representation of the structure of enoxaparin-loaded lipid hybrid nanoparticles. (**B**) The size distribution of lipid–polymer hybrid nanoparticle 2 (LPHN2). (**C**) The transmission electron microscopy (TEM) morphology of LPHN2. (**D**) In vitro release of profiles of enoxaparin from enoxaparin solution, enoxaparin-loaded solid lipid nanoparticles (SLNs), and enoxaparin-loaded LPHN2 (*n* = 3).

Double W/O/W emulsification technology was used to prepare enoxaparin-loaded lipid–polymer hybrid nanoparticles. Poloxamer 407 acted as the polymer core to load the drug. The schematic diagram of enoxaparin-loaded lipid–polymer hybrid nanoparticles is presented in Figure 1A. To screen the optimal amount of poloxamer 407, a control group (SLNs) was created with the absence of P407, and three different ratios (20%, 30%, 40%, *w/v*) of poloxamer 407 were tested. Their size and encapsulation efficiency are summarized in Table 1. The tested amount of P407 had no significant influence on particle size, polydispersity index, or zeta potential, but it led to different encapsulation efficiencies for enoxaparin. When the amount of P407 was set as 30%, the enoxaparin-loaded LPHN possessed a higher encapsulation efficiency of 65.72%. The results suggest that the addition of an appropriate concentration of P407 into the double emulsion could improve the encapsulation efficiency of enoxaparin. The higher encapsulation efficiency of LPHN2 could possibly be attributed to its appropriate viscosity. P407 is a thermo-sensitive polymer, and it can form gels when the ambient temperature is above gelation temperature. According to previous research [3,24,29], the increase in P407 concentration in the gel increases its viscosity. When the amount of P407 is 20%, its viscosity is not enough to restrain the enoxaparin in the internal gel core. Theoretically, the addition of 40% P407 could offer the highest encapsulation efficiency. However, the gel formed by 40% of P407 is too viscous to be dispersed well by ultrasonication, leading to lower encapsulation efficiency. Hence, LPHN2 was selected as the formulation in the following experiments. To investigate if the encapsulation efficiency of LPHN2 was changed with time, we tested the encapsulation efficiency after storage at 4 °C for one week. The encapsulation efficiency of LPHN2 stored at 4 °C for one week was 64.01%, which indicated that the encapsulation efficiency of LPHN2 could be kept unchanged for at least one week.

The average size of LPHN2 was about 150 nm, with a low PI (<0.30) (Figure 1B). In addition, there is a size distribution ranging from 10 to 100 nm in Figure 1B, which may be caused by the formation of Tween 80-based micelles in nanoparticle suspensions. The zeta potential of LPHN2 was slightly negative (−14.71 mV), which may be attributed to negatively charged egg lecithin in the surface of the LPHN.

Transmission electron microscopy (TEM) has been extensively used to observe the surface morphology of nanoparticles. The TEM image of LPHN2 is shown in Figure 1C, indicating that LPHN was spherical and about 150 nm in size, consistent with dynamic light scattering results.

The release of enoxaparin from LPHN2 was evaluated in pH 6.8 phosphate buffer and compared with the in vitro release of 0% P407-prepared SLNs and enoxaparin solution. The in vitro release profiles of enoxaparin solution, SLNs, and LPHN2 are shown in Figure 1D. Almost 98% of the drug released from the enoxaparin solution was within 10 h, which indicates that enoxaparin can diffuse freely through the dialysis bag. In contrast with the SLNs, there was a controlled and sustained release of enoxaparin from the LPHN2. Approximately 87% of the cumulative amount of enoxaparin was released from SLNs within 24 h. In the case of LPHN2, the percentage cumulative release of enoxaparin was about 61% within 24 h. The in vitro release result indicated that the incorporation of 30% P407 could control and sustain the enoxaparin release from LPHN compared with free P407 SLNs. There are two reasons to explain why LPH2 exhibited sustained release behavior in contrast with traditional lipid nanoparticles (SLNs). On the one hand, LPHN2 has higher encapsulation efficiency. For most of the drugs, they should diffuse from the nanoparticles first, and then release into the medium. Therefore, less amounts of free drugs could be released from the nanoparticles. On the other hand, the viscous gel core may delay drug diffusion from nanoparticles into the release medium.

### 3.2. Intestinal Absorption

**Figure 2:**
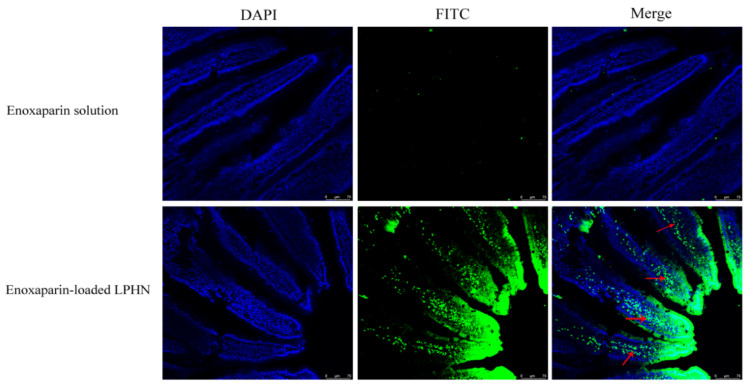
The LCSM images of the jejunum of rats at 0.5 h after administration of FITC-labeled (green) enoxaparin solution (the upper) and LPHN2 (the bottom). Cell nuclei of the jejunum sections were stained by DAPI (blue).

The absorption of enoxaparin-loaded LPHN2 in the intestine of rats was visualized by LCSM after oral administration. Figure 2 shows the intestine fluorescence signals after the intragastric administration of FITC-labeled enoxaparin solution and LPHN2. The LCSM images suggest that a more intense fluorescence was observed in the intestine after administration of FITC-labeled enoxaparin-loaded LPHN2 in comparison with the administration of FITC-labeled enoxaparin solution. In addition, the fluorescence signal can be viewed under a layer of intestinal epithelial cells, as indicated by the red arrows. Therefore, the LCSM results indicate that the drug in LPHN2 was not only accumulated in the surface of intestinal villi but had also penetrated the underlayer of intestinal epithelial cells.

### 3.3. In Vivo Pharmacokinetic Study in Rats

**Figure 3:**
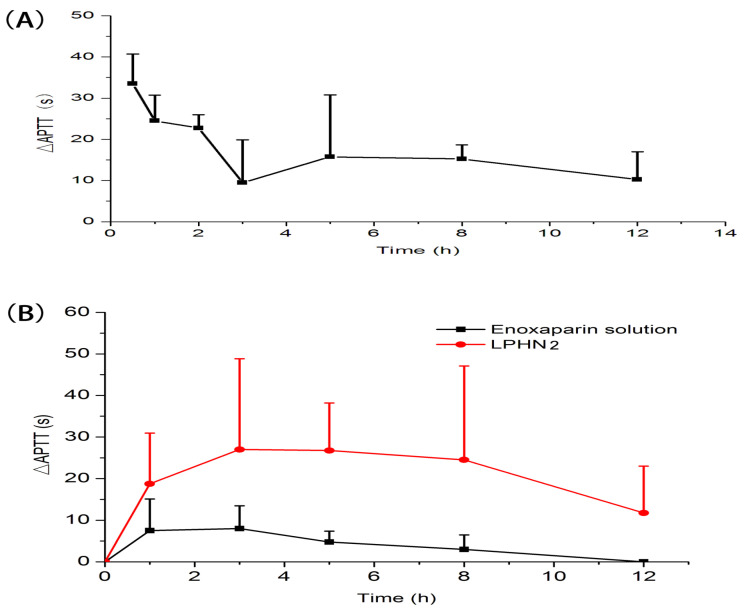
Mean Δ activated partial thromboplastin time (APTT) over time after a single intravenous injection of enoxaparin solution at a dosage of 101 IU/kg (**A**), and oral administration of enoxaparin solution and enoxaparin-loaded LPHN2 at a dosage of 1010 IU/kg (**B**) (*n* = 4).

In vivo pharmacokinetic behavior of enoxaparin-loaded LPHNs was investigated by measuring APTT in rats after intragastric administration, as shown in Figure 3. The main pharmacokinetic parameters are summarized in Table 2. The absolute bioavailability (F_abs_) of enoxaparin-loaded LPHN2 was 14.2%, a 6.8-fold increase compared with enoxaparin solution. The results indicate that the oral bioavailability of enoxaparin could be improved by lipid–polymer hybrid nanoparticles. As we know, the basic mechanisms that nanoparticles could improve oral absorption of drugs are as follows [30,31]: (1) encapsulation of drug to avoid degradation in the gastrointestinal tract before reaching the absorption site; (2) improving intestinal epithelial cell uptake. For drugs that are unstable in the gastrointestinal tract, high encapsulation efficiency is the precondition of oral absorption enhancement by nanoparticles. There are several reasons that could explain why LPHNs enhance the oral absorption of enoxaparin. On the one hand, LPHNs have higher encapsulation efficiency to protect enoxaparin from degradation in the stomach. On the other hand, on the basis of the intestinal absorption of LPHNs, LPHNs could overcome the mucus layer to facilitate enoxaparin entry to under layer of intestinal epithelial cells, followed by absorption into the systemic circulation.

Despite the results that LPHNs can improve the oral bioavailability of enoxaparin, its absolute bioavailability is still not high enough. There are several reasons that may explain this: (1) The encapsulation efficiency of enoxaparin in the LPNH is about 65%, and almost 35% of enoxaparin is a free drug in LPHN dispersion, and free enoxaparin usually has low oral bioavailability; (2) the nanoparticles must overcome the mucus layer before they are transported across the epithelium. Although one part of the nanoparticles can penetrate the mucus layer and be transported across the epithelium, other parts of the nanoparticle may be trapped in the mucus layer and eliminated from the gastrointestinal tract, owing to the mucus layer being renewed every 1~2 h [32]. Therefore, more rationally designed nanocarriers with higher encapsulation efficiency and stronger mucus layer permeability are needed to further improve the absolute oral bioavailability of enoxaparin.

### 3.4. In Vivo Efficacy

In vivo prevention of pulmonary thromboembolism of nanoparticles was studied in mice. As shown in Table 3, the inhibition effect was 58.3% when enoxaparin solution was intravenously administered. The inhibition effect of enoxaparin-loaded LPHN2 was 50.0%, 2.99-fold higher than that of enoxaparin solution after oral administration, which further indicated that lipid–polymer hybrid nanoparticles are effective in improving oral absorption and the inhibition effect of enoxaparin against thrombin-induced thrombosis.

### 3.5. Safety Evaluation

**Figure 4:**
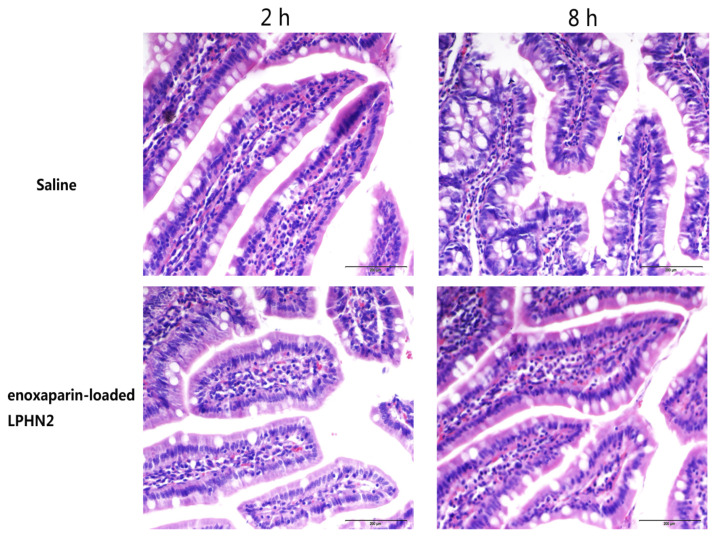
Morphology of intestinal mucosa of rats after oral administration of physiological saline- and enoxaparin-loaded LPHN2 after 2 and 8 h. Scale bar: 200 μm.

To investigate whether nanoparticles cause intestinal membrane damage or not, a histopathological examination was conducted. The results of pathological sections are shown in Figure 4. The histological studies indicated that there were no significant changes in the morphology and structure of the intestine exposed to enoxaparin-loaded LPHN2. The mucosal erosions and disruption of the enterocytes did not appear. Hence, LPHN is biocompatible in vivo as well as safe for the oral delivery of enoxaparin.

## 4. Conclusions

In summary, lipid–polymer hybrid nanoparticles (LPHNs) were prepared by double emulsification technology. The concentration of poloxamer 407 was optimized to ensure the high encapsulation efficiency of enoxaparin. Compared with traditional lipid nanoparticles, LPHNs possess not only higher encapsulation efficiency of enoxaparin, but also sustained release. In addition, optimized LPHNs could increase the concentration of enoxaparin in intestinal villi and facilitate enoxaparin penetration into the underlayer of enterocytes. Results of an in vivo pharmacokinetic study and an in vivo efficacy study further confirmed the superiority of LPHNs with regard to absorption-enhancing effects. In conclusion, rationally designed LPHNs could be excellent nanocarriers for oral delivery of enoxaparin.

## Figures and Tables

**Table 1:** Characterization of enoxaparin-loaded lipid-polymer hybrid nanoparticles at 3 different ratios of poloxamer 407 (*n* = 3).

Amount of P407	Size (nm)	PI	ζ Potential (mV)	EE (%)
0% (SLNs)	159.40 ± 1.59	0.293 ± 0.010	−21.83 ± 3.94	43.21 ± 3.79
20% (LPHN1)	149.70 ± 1.71	0.264 ± 0.030	−17.47 ± 1.20	43.14 ± 7.52
30% (LPHN2)	149.75 ± 2.45	0.293 ± 0.009	−14.71 ± 1.93	65.72 ± 14.33
40% (LPHN3)	153.19 ± 0.79	0.274 ± 0.002	−20.04 ± 1.59	59.47 ± 11.66

PI, polydispersity index; ζ potential, zeta potential; EE, encapsulation efficiency.

**Table 2:** Main pharmacokinetic parameters after peroral administration of enoxaparin formulations in rats at a dosage of 1010 IU/kg (*n* = 4). * *p* < 0.05 represents a significant improvement in absolute bioavailability in comparison with enoxaparin solution (p.o.).

Formulations	T_max_ (h)	AUC_0–12 h_(s·h)	F_abs_ (%)
Enoxaparin solution (i.v.)	-	182.3 ± 44.2	100.0
Enoxaparin solution (p.o.)	0.5	37.6 ± 10.0	2.1
Enoxaparin-loaded LPHN2 (p.o.)	3	258.3 ± 93.1	14.2*

i.v. means intravenous injection; p.o. means peroral administration.

**Table 3:** Inhibition effect of pulmonary thromboembolism by orally administered various enoxaparin formulations (*n* = 12).

Formulations	Inhibition Effect (% protection)
Saline (i.v.)	8.3
Enoxaparin solution (i.v.)	58.3
Enoxaparin solution (p.o.)	16.7
Enoxaparin-loaded LPHN2 (p.o.)	50.0

i.v. means intravenous injection; p.o. means peroral administration.
